# ACTH Stimulation Induced Self-Mutilation Behavior in the Golden Conure (*Guaruba guarouba*)

**DOI:** 10.3390/ani10030418

**Published:** 2020-03-02

**Authors:** Juliana Anaya Sinhorini, Cristiane Schilbach Pizzutto, Rupert Palme

**Affiliations:** 1 Independent Researcher, 05616-100 São Paulo, Brazil; jusinhorini@yahoo.com.br; 2 Department of Animal Reproduction, Faculty of Veterinary Medicine and Animal Science, University of São Paulo (USP), 05508-270 São Paulo, Brazil; crissp@usp.br; 3 Department of Biomedical Sciences, Unit of Physiology, Pathophysiology and Experimental Endocrinology, University of Veterinary Medicine, Veterinärplatz 1, 1210 Vienna, Austria

**Keywords:** corticosterone metabolites, non-invasive monitoring, abnormal behavior, feather picking

## Abstract

**Simple Summary:**

Psittacidae are very susceptible to chronic stress and behavioral disorders. We report a successful physiological validation of an enzyme immunoassay for the non-invasive monitoring of adrenocortical activity and, thus, stress in the golden conure. In addition, as an incidental finding, we demonstrate a link between increased glucocorticoid levels and behavioral disorders. Our results are of great relevance for conservation projects and endocrine-behavioral studies of captive golden conures, where the stress evaluation is a fundamental part of animal welfare programs.

**Abstract:**

Psittacidae are very susceptible to chronic stress and behavioral disorders. Information regarding the endocrinology of the golden conure is scarce, especially about adrenocortical activity. Endocrine studies using non-invasive methods are useful, because they allow longitudinal analysis with high numbers of samples without causing additional stress and are viable in the psittacidae management. The objective was to physiologically validate an enzyme immunoassay for measuring glucocorticoid metabolites (GCMs) in this species. Serial droppings were collected from 16 animals. First, one subgroup received adrenocorticotropic hormone (ACTH; Synacthen Depót^®^) and the other group served as controls. This procedure was reversed afterwards. All birds presented self-mutilation approximately 6 h after the ACTH injection. This behavior disappeared after two days. Peak concentrations (on average nine times higher than baseline values) of GCMs were found 6 (4–8) h after ACTH administration; in all (but one) animals also a second peak was found 14 (10–20) h post injection. GCM levels returned to the baseline after 24 h. We physiologically validated a cortisone enzyme immunoassays to measure adrenocortical activity in the golden conure. Such non-invasive methods are important for studies, which are related to welfare, reproductive, and conservation programs. In addition, we could demonstrate a link between increased glucocorticoid levels and behavioral disorders.

## 1. Introduction

The golden conure is an endemic species from the Brazilian rain forest. It is found in the extreme west of Maranhão but also in the north of Rondonia and the south of the Amazon River. The distribution of the species partially overlaps the Amazon “deforestation arch”. Over the last few years, the species lost at least 35% of its natural habitat [1] and is classified as vulnerable by the IUCN (Red list of Threatened Species-2017). The order Psittaciformes has been the target of many endocrine investigations, especially regarding stress and adrenocortical activity [2,3,4,5]. Understanding the effects of increased adrenocortical activity is of great relevance for reproductive, welfare and endocrine-behavioral programs, especially for captive animals [6,7,8].

Quantification of plasma hormones provides valuable information about the endocrine conditions of an animal. However, episodic fluctuations may occur, and these concentrations are also subject to daily and annual rhythms. Moreover, blood collection is a stressful event and can cause, on its own, hormonal alterations [9,10]. Monitoring urofecal steroid metabolites offers an alternative for the study of endocrine activity in birds, because it is a non-invasive and non-stressful method [10,11,12]. Moreover, this technique presents other advantages, such as ease in obtaining samples, possibility of subsequent collections for a long period of time without affecting the behavior of the studied animals and episodic hormone fluctuations that occur in the blood are smoothened in the feces [12,13].

The application of non-invasive methods for hormone monitoring has increased substantially in mammals and birds [12]. Studies have shown that in some bird species the determination of hormone metabolites in excrements is viable for sexing [14,15,16], measuring stress intensity [17,18] and gonadal activity [11,18,19,20,21]. However, expressed species differences in excreted metabolites exist [22]. Metabolites may also be present in conjugated form (sulfates or glucuronides), with greater or lesser affinity for antibodies and in some cases, previous treatment of the samples is necessary (e.g. hydrolysis of conjugated metabolites; [23]). At least some of these metabolites must be recognized by the antibody of an immunoassay to allow a measurement [22,23,24]. Whatever method is used to measure urofecal metabolites, it is mandatory to perform physiological and/or biological validations for each species and even both sexes, when of interest [12].

Thus, the objective of this study is to physiologically validate an enzyme immunoassay for measuring glucocorticoid metabolites (GCMs) in droppings of this species. In addition, as an incidental finding, we demonstrate a link between increased glucocorticoid levels and behavioral disorders.

## 2. Materials and Methods

### 2.1. Ethics Statement

This study has been authorized by SISBIO—(Sistema de Autorização e Informação em Biodiversidade) of the Chico Mendes Institute for Biodiversity Conservation—ICMBio, under number 344191-1 and approved by the Ethics Committee of the School of Veterinary Medicine and Animal Science, University of São Paulo, under protocol number 2823/2012 (28/11/2012).

### 2.2. Animals and Husbandry

For this study, 16 adult and clinically healthy golden conures (*Guaruba guarouba*) were used (8 males and 8 females). They belonged to the Scientific Breeder Lymington Foundation, located in Juquitiba/SP, Brazil. The birds were kept in stainless steel cages of 1 m height and 1 m^2^ area, without a nest box and divided in half in order to allocate one individual of each sex in each part. The aviaries had a daily cleaning/disinfection management and food/water changed twice a day (morning and twilight). All birds had water *ad libitum*. The food provided in the morning was composed of commercial extruded feed (Nutrópica^®^, Indaiatuba, SP), small germinated sunflower seed, varied fruits, vegetables and greens and a slice of green corn cob (2 cm) for each bird three to four times a week. Once a week, boiled egg, wheat germ and vitamin/protein complex (Aminomix pet^®^, Vetnil, Louveira, SP, Brazil) were added. In the afternoon, all birds received the same extruded commercial feed, mix of Psittacidae seed (canary grass, millet, niger, rapeseed, rice and oat) and varied fruits. 

To determine the reproductive period at the experimental site, a data analysis of the historical record of the Lymington Foundation was performed. Egg posture of the species occurred between January and March in Brazil, determining the reproductive period of the species in the foundation. 

### 2.3. Experimental Procedure

#### 2.3.1. Physiological Validation Experiment (ACTH Challenge Test) 

The group of golden conures was divided into two subgroups: Subgroup 1 (3 males and 3 females) and Subgroup 2 (5 males and 5 females). First, Subgroup 1 received an intramuscular injection of 0.5 mg/kg [2] of synthetic adrenocorticotropic hormone (ACTH; Tetracosactide, Synacthen Depót^®^, Novartis Pharma, Brussels, Belgium) into the pectoral muscle and Subgroup 2 was left untreated (controls). After 15 days, Subgroup 2 was ACTH injected (see above) and the other group served as control (untreated). Animals of the two sub-groups did not have visual or auditory contact with the other group during the experiments.

Bird droppings were collected 24 h before the ACTH administration (−24 h) to determine the baseline values. On the day of the injection, a collection was performed between 9:00 and 11:00 a.m., considered as 0 h. At 11:00 a.m. birds were physically constrained and ACTH was injected. Afterwards pooled samples were collected every 2 h over a 24-h period. After another day, a last sample collection was performed between 9:00 and 11:00 (+ 48h) a.m. All excrements were collected in plastic tubes, labelled, and stored in a −20 ℃ freezer until extraction.

#### 2.3.2. Steroid Extraction and Quantification

The homogenized droppings were lyophilized. Hormone metabolites were extracted according to the technique described by Palme et al. [25]. Briefly, after homogenization, an aliquot of 0.2 grams was weighted and 5 mL of 60% methanol added and shaken for 30 min. After centrifugation, 1 mL of the supernatant was transferred into plastic tubes for evaporation. The tubes containing the metabolites were sent to the University of Veterinary Medicine, Vienna, Austria. GCMs were quantified with a cortisone enzyme immunoassay (measuring GCMs with a 3,11-dioxo structure) as previously described [24]. 

### 2.4. Statistical Analysis

Baseline GCM concentrations were calculated by an iterative approach (see [12,26]) from all samples of an animal’s control treatment. We considered the assay physiologically validated when peaks in GCM concentrations could be detected after the ACTH administration. GCM values exceeding the mean plus 2 SD of the baseline were considered as peaks. 

The obtained data were tested for distribution normality and homogeneity of variances through the Kolmogorov-Smirnov test and the Bartlett test. If the data were considered normal and homogeneous, the One Way ANOVA test was used, followed by the Tukey post-test (*p* < 0.05). If there was no normality and homogeneity of the variances, the data were transformed (base 10 or X square root logarithm) and, if the transformation was not enough, non-parametrical analysis were performed, such as the Kruskal-Wallis (non-parametric ANOVA) followed by the Dunn (*p* < 0.05) or Mann Whitney (*p* < 0.05) tests. All statistical analyses were performed using the program GraphPad InStat 3.01, 32 bit for Windows^®^ 95 version. 

## 3. Results

All 16 birds pulled out their feathers and presented polyuria about 6 h after the ACTH administration (Synacthen Depót^®^). When we first observed these changes, we started the monitoring with instantaneous sampling (one/zero) every hour until they ended. The normalization of polyuria and the end of the feather pullout was observed 15–20 h and two days after the injection, respectively. Three birds presented blackened stools during a period of 6 to 20 h after administration. 

**Figure 1:**
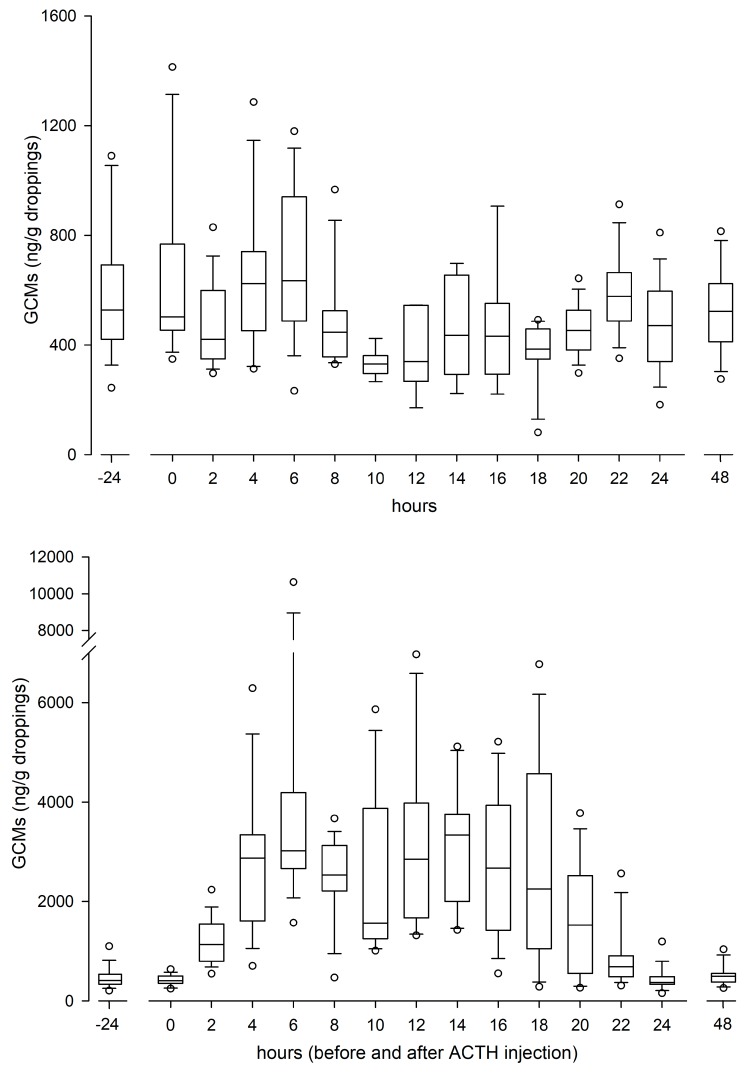
Box-plot graphs of concentrations of glucocorticoid metabolites (GCMs) in golden conure (*Guaruba guarouba*) excrements, in undisturbed control (upper panel) and ACTH injected (lower panel; n = 16 each) birds. Note the different y-axes scales.

There were no differences in GCM concentrations between males and females, neither during the day before (controls) nor after the ACTH challenges. Thus, the GCM levels of all animals are presented together. Baseline GCM concentrations were 495 ± 106 ng/g (mean ± SD). Following ACTH injection, GCM concentrations started to increase in the first sample (2 h) and reached peak (on average nine times higher than baseline) concentrations 6 (4–8) h (median; min-max) after the administration (Figure 1). In all (but one) animal also a second peak was found 14 (10–20) h post injection, which was sometimes (n = 7) even higher than the first one. GCM levels returned to the baseline after 24 h. 

## 4. Discussion

We performed this study in the golden conure (*Guaruba guarouba*), with a high number of birds, to physiologically validate a non-invasive method for the evaluation of the adrenocortical activity by measuring glucocorticoid metabolites in excreta. 

Although the behavioral observations were not the objective of this study, the feather pullout was an incidental finding that occurred in all the birds involved in the ACTH challenge. Owen and Lane [27] found that corticosterone metabolite levels in grey parrots (*Psittacus erithacus*) with self-mutilation were four times higher than those of birds, which did not present such a behavior. Although not in birds, Pizzutto et al. [7] also observed that elevated concentrations of GCMs were directly related to different degrees of self-mutilation in chimpanzees. However, both studies were only correlational and thus could not determine if high glucocorticoid (GC) levels are responsible for this behavior. In our study, the injection of ACTH elevated GC levels and also caused this form of self-mutilation. For Lennox and Harrison [28], the golden conure is one of the species, which most commonly presents behavioral alterations related to self-mutilation. Thus, the ACTH administration and the resulting increase in GC levels might have been sufficient to initiate this behavior. According to Carsia and Harvey [29], the most effective stimulus for GC secretion in birds is an ACTH injection, which was most likely also responsible for the self-mutilation behavior. Pizzutto et al. [7] and Mason [30] affirmed that self-mutilation is related to chronic stress, and the high post ACTH glucocorticoid levels in the golden conure may have mimicked such a condition.

In vertebrates, an ACTH administration simulates the natural response of the adrenals to stressful stimuli, causing a rapid raise of circulating GC (cortisol or corticosterone) concentrations within a few minutes [31]. The same pattern occurs in the droppings, but the elevation is delayed when compared to serum levels. However, there is a species specific variation in those delay times [12,32]. GCM levels started to increase in the first sample after injection and reached individual peak concentrations around 6 (main peak) and 14 h (second peaks) after the ACTH administration. Based on the pre-established criteria for the peak determination, a second significant peak in GCM concentrations occurred in all but one animals. This second peak was in 7 animals even higher than the first one. 

A study with parrots [3] reported that GCM peaks occurred 5–9 h after the ACTH administration. This delay in excretion corresponds with our findings (4–8 h). Rettenbacher et al. [24] also verified the presence of two peaks after the ACTH challenge in chicken and suggested that they represent urinary and fecal excretion, respectively. In birds, due to the simultaneous excretion of feces and urine, it is expected to find two GCM peaks, the first one reflecting predominantly the urinary excretion, and the second reflecting mainly the fecal portion [23], with a delay in relation to the first peak due to the intestinal time passage [24]. Individual variations in GC(M) concentrations have also been reported in several studies [10,31,33,34]. Therefore, Ferreira et al. [3] reinforced the necessity for longitudinal studies in which each individual serves as its own control, which makes the utilization of a non-invasive monitoring method even more important. The utilized cortisone EIA has also been successfully validated in various other bird species [2,3,35,36,37] and can be considered a powerful tool to evaluate husbandry and welfare conditions in birds.

## 5. Conclusions

On the basis of a successful physiological validation in the golden conure, we demonstrated that droppings can be used for the non-invasive monitoring of adrenocortical activity by measuring GCMs with a cortisone EIA. In addition, as an incidental finding, we could demonstrate a link between increased glucocorticoid levels and behavioral disorders. Our results are of great relevance for conservation projects and endocrine-behavioral studies of captive golden conures, where stress evaluation is a fundamental part of animal welfare programs.
